# Molecular characterization of non‐polio enteroviruses isolated from acute flaccid paralysis patients in Uganda

**DOI:** 10.1002/jmv.26804

**Published:** 2021-02-09

**Authors:** Phionah Tushabe, Wayne Howard, Josephine Bwogi, Molly Birungi, James P. Eliku, Proscovia Kakooza, Henry Bukenya, Prossy Namuwulya, Joseph Gaizi, Mayi Tibanagwa, Theopista Kabaliisa, Julius Mulindwa, Dennis Muhanguzi, Melinda Suchard, Nicksy Gumede, Barnabas Bakamutumaho

**Affiliations:** ^1^ EPI Laboratory Uganda Virus Research Institute Entebbe Uganda; ^2^ National Institute for Communicable Diseases Johannesburg South Africa; ^3^ World Health Organization Kampala Uganda; ^4^ Department of Biochemistry and Sports Sciences, College of Natural Sciences School of Biological Sciences, Makerere University Kampala Uganda; ^5^ Department of Biomolecular Resources and Biolaboratory Sciences College of Veterinary Medicine, Animal Resources and Biosecurity, Makerere University Kampala Uganda; ^6^ University of Witwatersrand Johannesburg South Africa; ^7^ WHO Regional Office for Africa Brazzaville Congo

**Keywords:** acute flaccid paralysis, Non polio enteroviruses, residual paralysis, semi‐nested PCR

## Abstract

Enteroviruses (EVs) are RNA viruses that can cause many clinical syndromes including acute flaccid paralysis (AFP). Within the global polio laboratory network, EVs are categorized either as polioviruses or non‐polio enteroviruses (NPEVs). Specific NPEVs have been described in polio‐like residual paralytic events in AFP patients. Retrospective analysis of 112 NPEV isolates from AFP patients was performed and thirty one NPEV types were identified of which 91% were *Enterovirus B* and 9% were *Enterovirus A* species. The NPEVs were distributed across the country with most patients in the eastern region (41/89; 46.1%). The highest proportion of patients were children less than 5 years (77/89; 86.5%) and male patients were more common (54/89; 60.7%). Echovirus 11 (11/89; 12.4%) was frequently observed and phylogenetic analysis of these sequences revealed high diversity. Coxsackievirus B5 (CV‐B5), CV‐B6, E21, and EV‐B69 were only seen in patients with residual paralysis. Analyses of the EV‐A71 sequence indicated a unique genogroup.

## 1 INTRODUCTION

Enteroviruses (EV) are non‐enveloped RNA viruses that primarily infect the gastro‐intestinal (enteric) tract. They belong to the family *Picornaviridae* and genus *Enterovirus*.^1^ This genus consists of fifteen species. Seven species (*Enterovirus A‐D* and *Rhinovirus A‐C* ^2^) are known to infect humans. EVs cause a spectrum of clinical conditions, including mild febrile illness, respiratory disease, acute flaccid paralysis (AFP), myocarditis, neonatal sepsis‐like illness and acute hemorrhagic conjunctivitis.^3^ Infections with non‐polio EV (NPEVs) are mostly asymptomatic, however, they can cause outbreaks in which small numbers of patients develop paralytic disease.^4^, ^5^, ^6^, ^7^ From 2014, parts of America and Europe have observed clustered cases of neurological illnesses (now termed acute flaccid myelitis) following outbreaks of respiratory illness due to EV D68 (EV‐D68).^7^, ^8^, ^9^ These observations suggest a causal relationship between NPEVs and paralytic disease.

EVs can undergo both inter and intra species recombination resulting into genetic divergence.^10^ Different EV types are classified based on sequence divergences in the capsid viral protein 1 (VP1) coding region.^11^ Conventionally, EVs are classified by the global polio laboratory network into two broad categories that is the polioviruses (e.g. wild polioviruses and vaccine derived polioviruses) and the NPEVs. NPEVs are prevalent globally, and in Africa and Uganda,^12^ they are often detected from patients with polio‐like residual paralysis (i.e. paralysis that does not resolve within 60 days after disease onset). Paralytic disease caused by polioviruses is at its lowest level in history but this chronic childhood disability might remain a public health burden if other risk factors, including NPEVs are unattended. We report on the findings of the molecular characterization of NPEVs prevalent in AFP patients in Uganda

## 2 METHODS

This was a descriptive retrospective cross‐sectional study that used routine surveillance (clinical and laboratory) data collected through the nation‐wide AFP surveillance program in Uganda for the period 2006 through 2016. Through this program, children below 15 years of age who present to health facilities with AFP are investigated to rule out polioviruses as a cause of paralysis. Clearance to use the surveillance samples and their accompanying data for this study was obtained from the Ministry of Health (Reference number ADM:322/462/01). Ethical approval was obtained from Uganda Virus Research Institute Research Ethics Committee (Reference number GC/127/18/03/617) and Uganda National Council for Science and Technology (Reference number HS 2396).

### 2.1 Sample collection and virus isolation

Samples were collected following AFP surveillance guidelines and virus isolation performed using L20B (a mouse cell line engineered to express the poliovirus receptor) and RD (cells derived from the human rhabdomyosarcoma) cell lines according to the standard WHO protocol for poliovirus isolation.^13^ Sixty (60) days after paralysis onset, senior clinical or medical officers conducted a patient follow‐up visit to establish presence or absence of residual paralysis for those patients whose samples were collected after 14 days from paralysis onset. At this visit, a detailed clinical neuralgic assessment was performed and a data capture form detailing these findings completed and forwarded to the National Polio Expert Committee (NPEC) for final classification of disease. Stool samples were included in this study only if an NPEV was detected following culture in RD and L20B cell lines and, follow up of the patient conducted after 60 days from disease onset to establish presence or absence of residual paralysis.

### 2.2 Enterovirus confirmation and FTA card preparation

A total of 10,806 stool specimens from 5417 patients suspected of AFP syndrome reported between 2006 and 2016 were investigated for evidence of enteroviruses. Only 1445 (13%) samples had NPEVs isolated from them, and of these, 132 (9%) cases had been followed up after 60 days from disease onset to confirm presence or absence of residual paralysis. Of the 132 cases, only 112 corresponding isolates were available and used in this study. To confirm presence of EVs in these isolates, a qualitative pan‐EV real‐time reverse transcription polymerase chain reaction (rRT‐PCR) of the 5′ untranslated region was performed in accordance with the Centers for Disease Control protocol.^14^ Briefly, each reaction mixture comprised of 1.0 µl primer/probe mix of EV specific primers and probe (Pan‐enterovirus S GGC CCC TGA ATG CGG CTA ATC C, Pan‐enterovirus A GCG ATT GTC ACC ATW AGC AGY CA, Pan‐enterovirus Probe (VIC) CCG ACT ACT TTG GGW GTC CGT GT), 10 µl of qScript XLT 1‐Step RT‐qPCR ToughMix (Quantabio), 8 µl PCR grade water and 1 µl of the isolate. The PCR conditions consisted of a reverse transcription reaction at 50°C for 30 min followed by an inactivation step at 95°C for 1 min and 40 PCR cycles of 95°C/15 s, 50°C/45 s and 72°C/5 s in an ABI7500 realtime thermocycler (Applied Biosystems). Following confirmation and for every sample, 200 µl of heat inactivated virus isolate was spotted onto labeled Flinders Technology Associates (FTA) cards (Whatman, Life Sciences) and air‐dried for 1 h in a biological safety cabinet. Dried FTA cards were individually packed into labeled pouches containing a dessicant and shipped at room temperature to the National Institute for Communicable Diseases (NICD), Johannesburg, South Africa for sequencing.

### 2.3 RNA extraction from FTA cards

Extraction of sample RNA from the FTA card samples was done according to the procedure developed by NICD. Briefly, twelve 4 mm discs were punched out of the FTA cards using a new biopsy disposable punch (Kai Europe) per card, and placed into two sterile and appropriately labeled 1.5 ml microfuge tubes (six discs per tube). A total of 200 µl of RNA processing buffer (80 µl of glycogen [5 mg/ml], 4 µl of 1 M dithiothreitol and 1.916 ml of Tris‐EDTA‐1 solution [10 mmol/L TRIS‐HCL/0.1 mmol/L EDTA, pH 7.6]) was added to the tubes containing the discs. The tubes were briefly vortexed and then heated at 70°C for 5 min. They were briefly spun and the supernatant transferred into another sterile microfuge tube containing 620 µl of buffer AVL mixed with carrier RNA. Viral RNA was then extracted using the QIAGEN QIAamp Viral RNA extraction kit (QIAGEN) following the manufacturer's guidelines^15^ and stored at −20°C until required for subsequent laboratory analyzes. Sterile, unspotted FTA cards were punched out and extracted alongside the FTA discs as RNA extraction controls.

### 2.4 Sequencing of part of the VP1 region

A sensitive semi‐nested RT‐PCR (RT‐snPCR) was performed as described by Nix et al.^16^ For quality control, RNA from a sample whose genotype had already been identified as well as the RNA extraction controls were included as positive and negative controls respectively. Amplification products were separated and visualized on 1% ethidium bromide (10 mg/ml) stained agarose gels and sized against a 100 bp molecular weight marker (Lonza). The DNA was then purified using the QIAquick PCR purification kit (QIAGEN) following the manufacturer's guidelines. The purified DNA was diluted to a final concentration of 7.5 µg/µl and sequenced using a BigDye Terminator V3.1 ready reaction cycle sequencing kit (Thermo Fisher Scientific). In brief, for each 10 µl reaction mixture, 5 µl of the PCR product, 2 µl of Big Dye 5 × buffer, 2 µl of BigDye, and 0.08 µl of each of primers AN88 and AN89 at a final concentration of 0.08 µmol/L were mixed. Amplification was done at 96°C for 1 min and 25 PCR cycles of 96°C/10 s, 50°C/5 s, 60°C/4 m. When sequencing failed with primers AN88/AN89, it was repeated with primers AN232/AN233 under similar PCR conditions. The sequencing reaction products were cleaned using the BigDye XTerminator purification kit (Life technologies) following the manufacturer's guidelines and finally loaded onto a 3500xL genetic analyzer (Applied Biosystems).

### 2.5 Enterovirus strain identification and phylogenetic analysis

The partial VP1 sequences obtained were analyzed using sequencher version 4.10.1 (Gene Codes Corporation) and the enterovirus strains identified with the Basic Local Alignment Search Tool (BLAST) and GenBank.^17^ Classification was done based on the algorithm proposed by Oberste et al. where a partial VP1 nucleotide sequence identity score of at least 75% to any enterovirus prototype strain indicated type identity.^18^ The types obtained with BLAST were corroborated using the Enterovirus Genotyping Tool (v0.1, RIVM) developed by National Institute for Public Health and the Environment, Netherlands (RIVM).^19^ Determination of EV types was also demonstrated by construction of phylogenetic trees using the partial VP1 sequences obtained and the respective reference sequences. All study and reference sequences were aligned using the ClustalW alignment program within the Molecular Evolutionary Genetics Analysis V7 (MEGA7) software.^20^ Trees were inferred with the Maximum Likelihood method and the robustness of the nodes was tested with 1000 bootstrap replications and bootstrap support values > 80 are shown at the nodes. All partial VP1 sequences from this study were deposited in GenBank with the accession numbers MT661794‐MT661882.

### 2.6 Data management and analysis

Data on patients' district of onset, date of onset of paralysis, sex, age, NPEC classification and follow‐up results was extracted from the existing AFP surveillance database. These data were collected from 2006 through 2016 from 65 districts of Uganda. NPEV type data obtained following molecular typing of the archived samples were stored in excel format and analyzed using EpiInfo™ version3.3.2.^21^

## 3 RESULTS

One hundred and twelve (112) NPEV isolates, each representing an AFP patient were investigated. Thirty (30) patients had residual paralysis while eighty two (82) patients had no evidence of paralysis. One hundred and four (104/112, 92.8%) isolates were pan‐EV real‐time positive with cycle threshold (*C*_t_) values ranging from 20 to 38. Of these, 94 (90.4%) were positive for enteroviruses using the RT‐snPCR and 89 (94.7%) were successfully typed.

### 3.1 Prevalence and distribution pattern of NPEV types (2006–2016)

**Figure 1:**
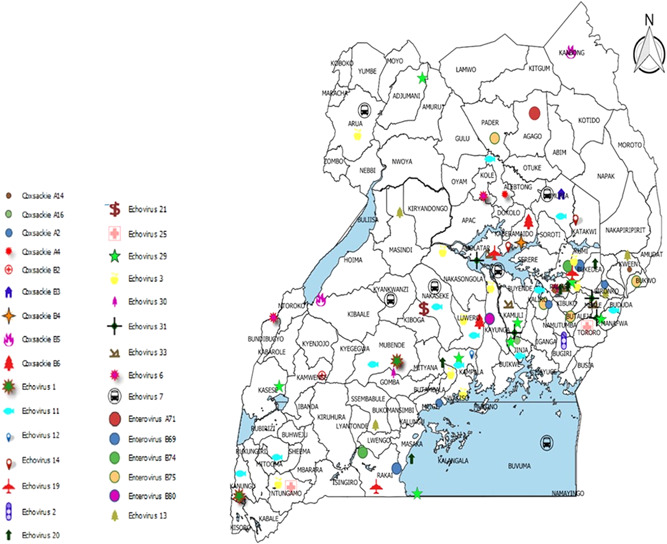
Distribution of NPEV types in Uganda for the period 2006–2016. NPEV, non‐polio enterovirus

Thirty one (31) NPEV types were obtained with percentage sequence identity matches ranging from 78% to 100%. The NPEV strains were geographically spread across the country with no definitive pattern (Figure 1). The eastern districts of Uganda had the highest number of patients and types while districts in western Uganda had the least patients and types. The age group with the highest prevalence of NPEVs was less than 5 years (77/89; 86.5%), followed by ≥15 to <10years (11/89; 12.4%) and ≥10 to <15years (01/89; 1.1%). Male patients (54/89; 60.7%) were more commonly affected than female patients (35/89; 39.3%). The NPEVs were distributed across the years with no single type dominant, however 2009 had the highest number of patients and types observed. The table below illustrates the NPEV types observed and their distribution (Table 1).

**Table 1:** showing NPEV distribution by year

Non‐polio enterovirus type	Rank	Frequency	%	Number in cases with residual paralysis	Number in cases without residual paralysis	Years detected (number)^a^
Echovirus 11 (E11)	**1**	**11**	**12%**	**03**	**09**	**2009 (5), 2007 (2), 2012, 2014 (3)**
Echovirus 3 (E3)	**2**	**9**	**10%**	**00**	**09**	**2007, 2009 (4), 2013 (2), 2015, 2016**
Echovirus 29 (E29)	**3**	**8**	**9%**	**04**	**04**	**2009 (2), 2012, 2013, 2014 (4)**
Echovirus 7 (E7)	**4**	**6**	**7%**	**02**	**04**	**2007, 2009 (3), 2012, 2016**
Echovirus 13 (E13)	5–6	5	6%	02	03	2007, 2009, 2014, 2015 (2)
Echovirus 20 (E20)	5–6	5	6%	01	04	2009 (4), 2014
Echovirus 33 (E33)	7–8	4	4%	00	04	2007 (2), 2010, 2013
Enterovirus B75 (EV‐B75)	7–8	4	4%	01	03	2009, 2010, 2011, 2014
Coxsackievirus A2 (CV‐A2)	9–11	3	3%	00	03	2006, 2007 (2)
Echovirus 19 (E19)	9–11	3	3%	00	03	2013, 2015 (2)
Echovirus 6 (E6)	9–11	3	3%	02	01	2009, 2012, 2013
Coxsackievirus A14 (CV‐A14)	12–19	2	2%	00	02	2007, 2015
Coxsackievirus B5 (CV‐B5)	12–19	2	2%	02	00	2009, 2014
Coxsackievirus B6 (CV‐B6)	12–19	2	2%	02	00	2010, 2011
Echovirus 1 (E1)	12–19	2	2%	00	02	2013, 2014
Echovirus 14 (E14)	12–19	2	2%	00	02	2006, 2009
Echovirus 25 (E25)	12–19	2	2%	01	01	2006, 2007
Enterovirus B69 (EV‐B69)	12–19	2	2%	02	00	2007, 2010
Enterovirus B74 (EV‐B74)	12–19	2	2%	01	01	2006, 2016
Coxsackievirus A16 (CV‐A16)	20–31	1	1%	00	01	2009
Coxsackievirus A4 (CV‐A4)	20–31	1	1%	00	01	2009
Coxsackievirus B2 (CV‐B2)	20–31	1	1%	00	01	2009
Coxsackievirus B3 (CV‐B3)	20–31	1	1%	00	01	2006
Coxsackievirus B4 (CV‐B4)	20–31	1	1%	00	01	2007
Echovirus 12 (E12)	20–31	1	1%	00	01	2011
Echovirus 2 (E2)	20–31	1	1%	00	01	2007
Echovirus 21 (E21)	20–31	1	1%	01	00	2014
Echovirus 30 (E30)	20–31	1	1%	00	01	2011
Echovirus 31 (E31)	20–31	1	1%	00	01	2009
Enterovirus A71 (EV‐A71)	20–31	1	1%	00	01	2014
Enterovirus B80 (EV‐B80)	20–31	1	1%	00	01	2006

Abbreviation: NPEV, non‐polio enterovirus.

^a^ Represents the number detected in a particular year. Where one NPEV was detected in a year, only the year is indicated.

Coxsackievirus B5 (CV‐B5), CV‐B6, E21, and EV‐B69 were isolated only from patients with residual paralysis while all CV‐A viruses isolated along with CV‐B2, CV‐B3, CV‐B4, E1, E12, E14, E19, E2, E3, E30, E31, E33, and EV‐B80 virus types were obtained from patients without residual paralysis. Echovirus 11 was the most frequently identified EV in this study (11/89; 12.4%). Echovirus 29 (4/24; 16.7%) was frequently detected in patients with residual paralysis and E3 (6/65; 9.2%) in those without residual paralysis.

### 3.2 Genetic diversity of NPEV types

**Figure 2:**
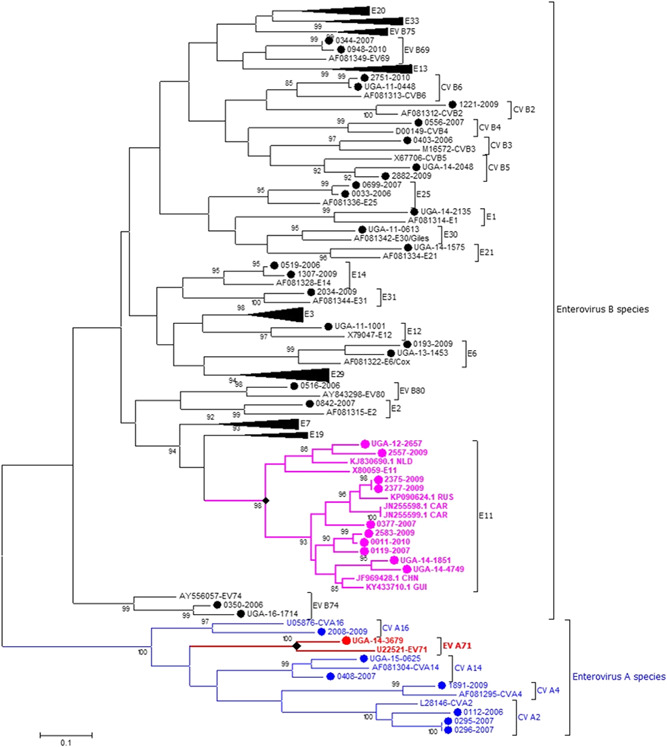
Phylogenetic tree showing NPEV types obtained (2006–2016) following amplification of part of the VP1 region. Study sequences are shown with a blue, red, purple or black dot; reference sequences are shown without a dot; E11 study sequences are shown with selected sequences from elsewhere; *Enterovirus B* types with 3 or more study sequences (except E11) are shown as a compressed subtree. Tree was inferred with the Maximum Likelihood method based on the General Time Reversible model as identified by the best DNA/protein models program in MEGA7. NPEV, non‐polio enterovirus

**Figure 3:**
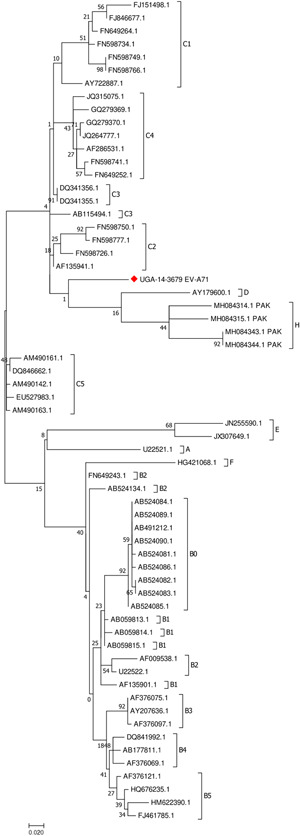
Phylogenetic tree of the sequence UGA‐14‐3679 with representative EV‐A71 sequences belonging to genogroups A–H and subgenogroups C1‐C5 and B0‐B5 as proposed by Bessaud et al.^22^ The sequence UGA‐14‐3679 is indicated with a red diamond. Tree was inferred with the Maximum Likelihood method based on the Kimura 2‐parameter model as identified by the best DNA/protein models program in MEGA7

Eighty one (81/89; 91%) of the observed partial VP1 sequences belonged to *Enterovirus B* species (Figure 2). Phylogenetic analysis of Echovirus 11 (E11) sequences from Uganda revealed high sequence diversity and clustered with those from West Africa, China, Russia and the Netherlands. Phylogenetic analysis of the EV‐A71 partial VP1 sequence along with sequences belonging to different genogroups (A–H) and sub‐genogroups (C1‐C5 and B0‐B5) (Figure 3) showed that the sequence UGA‐14‐3679 (MT661795) from Uganda did not cluster with any of the other sequences.

## 4 DISCUSSION

Enteroviruses can be commensal flora and the finding of an enterovirus in fecal samples is not necessarily causal. However, when paralysis is caused by an enterovirus, fecal samples are a useful material for viral detection as the virus can be shed in feces for weeks to months following disease onset. Global surveillance for pathogenic enteroviruses can guide future vaccine development and control strategies.

Enterovirus‐related infection especially due to specific strains among children diagnosed with polio‐like paralysis and who test negative for polioviruses, may explain the sequel that clinically manifests with chronic (residual) neurologic disability. Data from this study indicated that majority of NPEVs belonged to EV‐B similar to cell culture studies elsewhere.^23^, ^24^, ^25^, ^26^, ^27^ Most of these studies have used only two susceptible cell lines (RD and L20B cell lines) as well as a small number of cell culture passages for the NPEV isolation process. The WHO Regional office for Europe enterovirus surveillance guidelines recommend use of a minimum of three susceptible cell lines to improve the chances of successful virus isolation.^28^ Most of the NPEV studies that have been done in WHO specialized polio laboratories use only two cell lines (RD and L20B cell lines) for EV isolation because the surveillance targets poliovirus and not other EVs. The L20B cell line is highly sensitive and selective for polioviruses,^29^ however, this mouse cell line does only grow a limited number of NPEVs.^30^ Previous studies of enteroviruses cultured using the L20B and RD cell lines have shown a high predominance of EV‐B species.^25^, ^26^, ^27^, ^30^, ^31^, ^32^, ^33^ These findings are suggestive of cell culture bias towards EV‐B species, however, it should be noted that there are 63 known EV‐B types, versus 25 EV‐A, 23 EV‐C, and 05 EV‐D types and as such, EV‐Bs will often seem more common.

None of the NPEV types was predominant, similar to reports from West Africa and India.^24^, ^25^, ^26^ Despite this, E11 (11; 12.4%) was most frequently detected. E11 has previously been reported in AFP patients from Romania, the United States and India 34, 35, 36 and is said to be the most commonly isolated human enterovirus ^37^ with high genetic diversity.^38^ Phylogenetic analysis of the E11 sequences showed high diversity (Figure 2) with the study sequences clustering with sequences from West Africa, China, Russia and the Netherlands. Echovirus 29 (4; 16.7%) was the most frequently detected in the patients with residual paralysis while E3 (9; 13.8%) in patients without residual paralysis. All CV‐A viruses detected, along with CV‐B2, CV‐B3, CV‐B4, E1, E12, E14, E19, E2, E3, E30, E31, E33, and EV‐B80 virus types were only identified in patients without residual paralysis while CV‐B5, CV‐B6, E21 and EV‐B69 were only identified in patients with residual paralysis. CV‐B6 has previously been reported in two patients with residual paralysis in India.^26^ Male patients (54/89; 60.7%) were predominantly infected with NPEVs as was observed in Zambia (54.9%), Nigeria (63%), and Mongolia (56%).^23^, ^39^, ^40^ Enteroviruses were also predominant in children below 5 years, similar to reports from India.^26^ However, this may be due to higher prevalence or pathogenicity of enteroviruses in younger children or a bias towards inclusion of this age‐group in AFP surveillance programs.

Enterovirus A71 (EV‐A71) and CV‐A16 are common causes of hand, foot and mouth disease (HFMD), and have caused recurrent outbreaks in the Asia‐Pacific region since the 1990s. Phylogenetic analysis of the EV‐A71 sequence from Uganda suggested a unique genogroup that has not been reported before (Figure 3). Only seven (7) EV‐A71 genogroups (genogroups A–G) were previously recognized.^41^ Genogroups A (prototype BrCr strain) and B have not been detected recently, while genogroup C (lineages C1–C5) currently circulate in Asia, the United States, and Europe. EV‐A71 genogroups D and G are indigenous to India while E and F were reported in Africa.^41^ However, a new genogroup H has been reported in Pakistan.^42^ The divergent Ugandan EV‐A71 isolate (tentatively genogroup I) has not been associated with clusters of neurological disease or HFMD. Novel pathogens often have the potential to cause outbreaks or fatal disease outcomes hence the need for continued monitoring of NPEVs in Uganda.

### 4.1 Limitations and strengths of the study

The sample size was small but can contribute to future meta‐analyzes in which significance testing might establish a statistical correlation between residual paralysis and circulating NPEV types. The use of two cell lines as opposed to the recommended minimum of three ^28^ is another limitation of this study. In addition, expansion of enterovirus surveillance to include sewage as well as AFP samples will allow better understanding of the role of NPEVs in non‐polio AFP etiology and provide a more complete picture of the diversity of circulating NPEV types. As the term “acute flaccid paralysis” can sometimes encompass nonspecific syndromes resulting in weakness and immobility, the strength of our study is the availability of clinical data on presence and absence of residual paralysis lasting longer than sixty days. Enteroviruses associated with permanent paralysis are more urgent targets for diseases control and future vaccine development, although pooled data from multiple countries would be required.

## 5 CONCLUSION

This study revealed a high NPEV type diversity in patients clinically diagnosed with AFP. Some NPEV types were only observed in patients with residual paralysis. This facet of the study adds to the understanding of the natural history of such infections in Uganda and globally. The detection of EV‐A71, which is most commonly associated with HFMD but also associated with severe neurological disease (including permanent paralysis) and fatal outcomes (neurogenic pulmonary edema), calls for formulation of effective long term strategies to monitor NPEV circulation in Uganda.

## CONFLICT OF INTERESTS

The authors declare that they have no conflict of interest.

## AUTHOR CONTRIBUTIONS

PT: conceived the idea, designed the study, conducted laboratory analyses (sequencing), data analysis, drafted and reviewed the manuscript; WH: conducted laboratory analyses (sequencing), sequence data analysis and reviewed the manuscript; JB: designed the study, drafted and reviewed the manuscript; MB: data analysis and reviewed the manuscript; JPE: laboratory analysis (cell culture) and reviewed the manuscript; PK: data analysis and reviewed the manuscript; BH: laboratory analysis (cell culture) and reviewed the manuscript; PN: laboratory analysis (cell culture) and reviewed the manuscript; JG: laboratory analysis (cell culture) and reviewed the manuscript; MT: laboratory analysis (cell culture) and reviewed the manuscript; TK: laboratory analysis (cell culture) and reviewed the manuscript; JM: designed the study drafted and reviewed the manuscript; DM: designed the study, drafted and reviewed the manuscript; MS: drafted and reviewed the manuscript; NG: designed the study, drafted and reviewed the manuscript; BB: conceived the idea, designed the study, drafted and reviewed the manuscript.

## Data Availability

The data that support the findings of this study are available from the corresponding author upon reasonable request.
